# Anatomical, chemical and endophytic fungal diversity of a Qi-Nan clone of *Aquilaria sinensis* (Lour.) Spreng with different induction times

**DOI:** 10.3389/fpls.2024.1320226

**Published:** 2024-03-25

**Authors:** Xiaofei Li, Xiaoying Fang, Zhiyi Cui, Zhou Hong, Xiaojin Liu, Gaiyun Li, Houzhen Hu, Daping Xu

**Affiliations:** ^1^ Research Institute of Tropical Forestry, Chinese Academy of Forestry, Guangzhou, Guangdong, China; ^2^ College of Landscape Architecture, Nanjing Forestry University, Nanjing, Jiangsu, China; ^3^ Research Institute of Wood Industry, Chinese Academy of Forestry, Beijing, China

**Keywords:** *Aquilaria sinensis*, Qi-Nan, chemical composition, endophytic fungi, antioxidant activity

## Abstract

Recently, some new Qi-Nan clones of *Aquilaria sinensis* (Lour.) Spreng which intensively produces high-quality agarwood have been identified and propagated through grafting techniques. Previous studies have primarily focused on ordinary *A. sinensis* and the differences in composition when compared to Qi-Nan and ordinary *A. sinensis*. There are few studies on the formation mechanism of Qi-Nan agarwood and the dynamic changes in components and endophytic fungi during the induction process. In this paper, the characteristics, chemical composition, and changes in endophytic fungi of Qi-Nan agarwood induced after 1 year, 2 years, and 3 years were studied, and Qi-Nan white wood was used as the control. The results showed that the yield of Qi-Nan agarwood continued to increase with the induction time over a period of 3 years, while the content of alcohol extract from Qi-Nan agarwood reached its peak at two years. During the formation of agarwood, starch and soluble sugars in xylem rays and interxylary phloem are consumed and reduced. Most of the oily substances in agarwood were filled in xylem ray cells and interxylary phloem, and a small amount was filled in xylem vessels. The main components of Qi-Nan agarwood are also chromones and sesquiterpenes. With an increasing induction time, the content of sesquiterpenes increased, while the content of chromones decreased. The most abundant chromones in Qi-Nan agarwood were 2-(2-Phenethyl) chromone, 2-[2-(3-Methoxy-4-hydroxyphenyl) ethyl] chromone, and2-[2-(4-Methoxyphenyl) ethyl] chromone. Significant differences were observed in the species of the endophytic fungi found in Qi-Nan agarwood at different induction times. A total of 4 phyla, 73 orders, and 448 genera were found in Qi-Nan agarwood dominated by *Ascomycota* and *Basidiomycota*. Different induction times had a significant effect on the diversity of the endophytic fungal community in Qi-Nan. After the induction of agarwood formation, the diversity of Qi-Nan endophytic fungi decreased. Correlation analysis showed that there was a significant positive correlation between endophytic fungi and the yield, alcohol extract content, sesquiterpene content, and chromone content of Qi-Nan agarwood, which indicated that endophytic fungi play a role in promoting the formation of Qi-Nan agarwood. Qi-Nan agarwood produced at different induction times exhibited strong antioxidant capacity. DPPH free radical scavenging activity and reactive oxygen species clearance activity were significantly positively correlated with the content of sesquiterpenes and chromones in Qi-Nan agarwood.

## 1 Introduction

Agarwood, the resinous wood obtained from wounded *Aquilaria* trees, is utilized not only in pharmaceuticals for its exceptional medicinal properties (Ibrahim and Mohamed, 2015), but also extensively utilized in incense and perfume (Chen et al., 2016). When *A. sinensis* is exposed to external stimuli, such as physical or chemical damage or endophytic fungi, agarwood is formed (Putri et al., 2017). In natural forest environments, only a small percentage, specifically 7-10%, of *A. sinensis* trees can produce agarwood, and we can ‘t obtain agarwood from healthy *A. sinensis* (Lv et al., 2022). Therefore, the market demand for agarwood significantly exceeds its available supply (Al-Hindi et al., 2017). Due to widespread logging of wild agarwood trees, *A. sinensis* was included in the List of National Key Protected Wild Plants in 1999 and listed as a Class II endangered plant in China (National Key Protection of Wild Plants List, 1999). Now all *Aquilaria* species are listed in Appendix II of the Convention on International Trade in Endangered Species of Wild Fauna and Flora (CITES, 2005). Due to the reduction of wild trees, Hainan, Guangdong, Guangxi and some other regions began to cultivate *A. sinensis* plantations at the end of the last century (Tian et al., 2021).

In China, Qi-Nan agarwood has been considered as the representative of high-quality agarwood, and its elegant scent can be smelled without heating (Yu et al., 2021). In the process of finding high-quality agarwood, people have found a special agarwood germplasm of *A. sinensis* that can produce high-quality agarwood by cold drilling and is propagated by grafting on ordinary seed seedlings of *A. sinensis* (Yu et al., 2021) which is called Qi-Nan agarwood (QI-NAN), Chi-Nan agarwood (CNA)or Jar-Nan agarwood in China, Kanankoh or Kyara in Japan, and Tagara in India (Yang et al., 2014a). Compared to *A. sinensis*, this special kind of germplasm of *A. sinensis* more easily induces agarwood formation and yields high-quality agarwood (Zhang et al., 2022). For ordinary of *A. sinensis*, it is highly challenging to procure agarwood with an alcohol-soluble extract content exceeding 10% through the drilling method over a period of one year, but this unique germplasm of *A. sinensis*, Qi-Nan, can obtain, and the agarwood has more than 40% alcohol-soluble extract content, which is induced by the same drilling method (Yu et al., 2021). However, the number of studies on Qi-Nan is far less than that on ordinary *A. sinensis*, and most of the studies on Qi-Nan have focused on the difference in composition between Qi-Nan agarwood and ordinary agarwood (Yu et al., 2021; Lv et al., 2022).

The mechanism of Qi-Nan agarwood formation is still not fully comprehended, but it is quite clear that the primary components of agarwood from Qi-Nan are sesquiterpenes and phenylethyl chromones (Islam and Banu, 2021), and the principal sesquiterpenes constituents of *A. sinensis* agarwood include guaianes, eudesmanes, acoranes, eremophilanes, cadinanes, agarospiranes, zizaanes, humulanes and prezizaanes. These compounds are primarily found in plants, predominantly as volatile components within essential oils, and are characterized by distinct aromas that enhance the fragrance of agarwood (Li et al., 2020). 2-(2-phenylethyl) chromones are the primary constituents found in agarwood phenylethyl chromones. More than 100 derivatives of 2-(2-phenylethyl) chromones have been documented, with numerous exhibiting promising pharmacological properties such as neuroprotective, cytotoxic, antibacterial, acetylcholinesterase inhibitory, anti-inflammatory, and antioxidant activities (Wang et al., 2016). The study by Yu et al. (2021) revealed that Chi-Nan agarwood exhibited higher levels of the primary sesquiterpenes compared to ordinary agarwood, particularly in terms of guaiane and eudesmane derivatives. Moreover, the concentrations of 2-(2-phenylethyl) chromone and 2-[2-(4′-methoxyphenyl) ethyl] chromone were significantly elevated in this unique agarwood germplasm of *A. sinensis* in comparison to ordinary *A. sinensis* agarwood (Zhang et al., 2022).

Agarwood induction is a complex dynamic process (Liu J. et al., 2022). When the *Aquilaria* tree is damaged, microbial infection often occurs at the same time and will last for a long time. Therefore, many studies suggest that the formation of agarwood in *Aquilaria* trees is the result of the combined action of external damage and microorganisms, especially the endophytic fungi in *Aquilaria* trees (Chen et al., 2017). In this process, it was found that the carbohydrates in *A. sinensis* decreased whereas the related substances of agarwood increased (Liao et al., 2018), and these specific metabolites would promote plant wound healing and anti-microbial (Chen et al., 2021), while agarwood was formed.

The majority of systematic investigations into the dynamic alterations in secondary metabolites during agarwood formation have been centered on ordinary *A. sinensis*, and there are almost no studies on the Qi-Nan germplasm of *A. sinensis*. In this study, the agarwood constituents, endophytic fungi and antioxidant capacity were studied at 1, 2 and 3 years after induction for the purpose of revealing the dynamic changes in the process of agarwood formation. The composition and content of sesquiterpenes and chromophenoids, the infestation of endophytic fungi and the antioxidant capacity in agarwood of a Qi-Nan clone of *A. sinensis* at different periods were analyzed. It will be very helpful for people to understand Qi-Nan clones of *A. sinensis* clearly.

## 2 Materials and methods

### 2.1 Qi-Nan agarwood materials and experimental design

The Qi-Nan clone, named Xi Guaye because of its large, rounded, and dark green leaves, was produced by the Chunlong Qi-Nan nursery base (111.35.36 E longitude, 21.68.85 N latitude), Mata Town, Dianbai District, Maoming City, Guangdong Province and planted locally. We chose Xi Guaye as the test material, because of its fast formation of agarwood, high yield and high quality of agarwood produced, which is a representative clone among many Qi-Nan clones. Three-year-old healthy Qi-Nan trees with a DBH more than 4.5 cm were selected for the experiment. Every 15 trees were repeated once, and a total of 45 trees were repeated three times. Before drilling, three trees were randomly sampled from each replicate and this process was repeated three times to determine the endophytic fungi and ethanol extracts as a control group. The hole was drilled vertically on the same side of the tree, 10 cm above the grafting interface, using an electric drill. The pore size was 0.8 cm, the hole spacing was 8 cm, and the hole depth was approximately 3/4 of the diameter of the tree. After drilling, three trees from each replicate were randomly selected and harvested, and samples were collected at intervals of 1 year, 2 years, and 3 years to determine the yield. At the same time, endophytic fungi were sampled and determined on the stem of the Qi-Nan tree that was obtained.

### 2.2 Sample preparation

For the purpose of identifying endophytic fungi, 20 cm^3^ (2 cm × 2 cm × 5 cm) of fresh trunk center was taken from Qi-Nan trees before drilling and 1, 2, and 3 years after drilling and storing the samples in an ultra-low temperature freezer (-80°C).

To acquire agarwood, the uncolored portion of the wood, namely white wood, was extracted using a specialized agarwood knife, leaving behind the dark resinous portion as agarwood. Then the agarwood was dried in a cool, well-ventilated area and 20 g of Qi-Nan agarwood was taken and crushed by a crushing machine and passed through a 2 mm sieve for later use. In addition, he white wood in the central part of the trunk of Qi-Nan before drilling was taken as a control, and the same was dried and crushed for later use.

### 2.3 Agarwood yield and ethanol extract content

Following the collection of samples, the tree stems were allowed to dry by air and subsequently measured for weight. Similarly, white wood was removed, and the agarwood yield is the ratio of the weight of the air-dried agarwood to the amount of air-dried total wood (w/w % DW).

The ethanol extract concentration was determined through the utilization of an alcohol microwave oscillation extraction technique (Nguyen et al., 2021). 2 g dry white wood and Qi-Nan agarwood powder sample was taken into a volume flask, and 20 mL of 95% alcohol was added and extracted at 60°C and 750 W supersonic conditions (Elmasonic P300H, Germany) for 60 min. The ethanol extract underwent filtration using a 0.45 μm filter membrane and was then concentrated through rotary evaporation using a Concentrator 5301 from Eppendorf, Germany (Zhang et al., 2022). The ethanol extract content is the ratio of the weight of the ethanol extract to the weight of the agarwood powder (w/w % DW).

### 2.4 Xylem anatomical structure and histochemical staining

The wooden slices were transformed into cubic blocks measuring 1 × 1 × 1 cm3 and promptly preserved in a solution of FAA, consisting of a mixture of formaldehyde, glacial acetic acid, 95% ethanol, and distilled water in the proportions of 5:5:50:40 (Li et al., 2022). Subsequently, 20-micrometer thick slices were obtained from the specimens utilizing an ultramicrotome (Leica EM UC7, Germany). These slices were treated with iodine-potassium iodide (I2 - KI) for the identification of starch granules, while the detection of polysaccharide compounds was achieved through staining with Periodic acid-Schiff (PAS) reagent. The examination of all sections was conducted using a Nikon 80i light microscope (Nikon, Japan) (Liu Y. et al., 2022).

### 2.5 GC-MS analysis condition

Gas chromatography-mass spectrometry (GC-MS) (GCMS-QP2010 Plus, Shimadzu, Japan) was used to analyze the components of ethanol extract of Qi-Nan agarwood. GC conditions:

Gas chromatography was performed under specific conditions using a DB-5HT 5% Phenyl Methyl Siloxane column (30 m × 0.25 mm × 0.1 μm) (Agilent, Palo Alto, CA, USA) to separate samples. The starting temperature was kept constant at 90°C for a duration of 1 minute, followed by a gradual increase to 150°C at a rate of 2°C per minute, where it was sustained for 5 minutes. Subsequently, the temperature was further raised to 280°C at a rate of 2°C per minute and maintained for a period of 10 minutes. The inlet temperature was 250°C; the temperature of vaporization chamber was 250°C; the carrier gas utilized in the experiment was helium (He) with a purity of 99.999%; the flow rate of the carrier gas was maintained at 1.0 mL/min, with a split ratio of 20:1; the injection volume for the sample was 1 μL. MS conditions: chromatographic-mass spectrometry interface temperature 250°C; ionizing compounds through electron impact (EI) was conducted with an emission current of 70Ev and the ion source temperature of 230°C; the spectra were acquired across the mass range of m/z 50 ~ 500. The solvent was delayed for 5 min.

GC-MS Realtime Analysis (Shimadzu, Japan) was used to collect mass spectrometry information, and GC-MS Postrun Analysis (Shimadzu, Japan) was used for data processing, including peak extraction and area integral. According to the search results of the NIST (2014) standard mass spectrometry library and reference to relevant literature, the possible compound components were confirmed, and the relative content of each chemical component was calculated by the peak area normalization method (Wang et al., 2016).

### 2.6 Endophytic fungi determination

#### 2.6.1 DNA extraction and PCR amplification

The Qi-Nan samples underwent a disinfection process involving immersion in 75% ethanol for 1 minute, followed by exposure to 3.25% sodium hypochlorite for 3 minutes, another immersion in 75% ethanol for 30 seconds, and subsequently rinsed thrice with sterile distilled water to decontaminate the surface of the samples (Yao et al., 2019). Subsequently, 20 g samples were taken and frozen in liquid nitrogen to extract genomic DNA. 0.5 g sample was added to a 2 mL centrifuge tube, crushed and grinded (FastPrep-24 5G, MP, USA) at 45 HZ for 250 s, and then transferred to 5 mL CTAB extraction buffer, which was preheated to 65° C in advance. Genomic DNA was extracted from endophytic fungi using an improved CTAB method (Guo et al., 2000). DNA concentrations were determined using a NanoDrop2000 spectrophotometer (Thermo Scientific, USA).

The internal transcribed spacer 2 (ITS2) region of the endophytic fungal ribosomal RNA gene was amplified through PCR, involving a series of temperature cycles (95°C for 2 min, followed by 25 cycles at 95°C for 30 s, 55°C for 30 s, and 72°C for 30 s, with a final extension at 72°C for 5 min). The amplification process utilized primers ITS1F (5’-CTTGGTCATTTAGAGGAAGTAA-3’) and ITS2R (5’-GCTGCGTTCTTCATCGATGC-3’), with a unique eight-base barcode sequence assigned to each sample. PCR reactions were conducted in triplicate using a 20 μL mixture comprising 4 μL of 5 × FastPfu Buffer, 2 μL of 2.5 mM dNTPs, 0.8 μL of each primer (5 μM), 0.4 μL of FastPfu Polymerase, and 10 ng of template DNA. Subsequently, amplicons were isolated from 2% agarose gels and purified employing the AxyPrep DNA Gel Extraction Kit (Axygen Biosciences, Union City, CA, U.S.) following the manufacturer’s guidelines (Yao et al., 2019).

#### 2.6.2 Library construction and sequencing

The purified PCR products were quantified utilizing the Qubit^®^3.0 system (Life Invitrogen), and every 24 amplicons whose barcodes were different were mixed equally. The combined DNA samples were utilized for the construction of an Illumina Pair-End library, following the genomic DNA library preparation protocol provided by Illumina. Subsequently, the amplicon library underwent paired-end sequencing (2 × 250) on an Illumina MiSeq platform (Shanghai BIOZERON Co, Ltd) in accordance with established procedures. The raw sequencing data were archived in the NCBI Sequence Read Archive (SRA) database under the Accession Number: PRJNA1021371.

#### 2.6.3 Processing of sequencing data

The initial step involved demultiplexing the raw fastq files using in-house Perl scripts, which utilized the barcode sequence information specific to each sample. The demultiplexing process adhered to the following criteria: (i) Reads of 250 base pairs were truncated at any positions where the average quality score fell below 20 within a 10 base pair sliding window; any resulting truncated reads shorter than 50 base pairs were excluded. (ii) Stringent criteria were applied, including exact barcode matching, allowance for a 2-nucleotide mismatch in primer matching, and the removal of reads containing ambiguous characters. (iii) Sequences with overlapping regions exceeding 10 base pairs were assembled based on their overlapping sequences. Reads that could not be successfully assembled were eliminated from further analysis.

The operational taxonomic units (OTUs) were clustered with a 97% similarity threshold utilizing UPARSE version 7.1 (http://drive5.com/uparse/), and chimeric sequences were detected and eliminated through UCHIME (Sun et al., 2023). The phylogenetic affiliation of each gene sequence was analyzed by RDP Classifier (http://rdp.cme.msu.edu/) against the UNITE database version 18.11.2018 using the sintax function in USEARCH with a confidence cut-off (P) value of 0.65 (Yao et al., 2019).

#### 2.6.4 Phylogenetic reconstruction

The phylogenetic tree was constructed using the top ten endophytic fungi isolated from Qi-Nan. The fungal sequences of interest were uploaded to the NCBI database (https://www.ncbi.nlm.nih.gov/) and compared with known fungal sequences. The resulting BLAST sequence file was then downloaded. We obtained a set of 14 sequences, consisting of 13 similar sequences, which were subsequently used to construct a phylogenetic tree using MEGA 7 software (Kumar et al., 2008).

### 2.7 Antioxidant capacity

#### 2.7.1 DPPH radical scavenging activity assay of alcohol extract

A solution of DPPH (3.5 mg) was prepared by diluting it with ethanol in a 100-mL volumetric flask of brown color, resulting in a final volume of 100 mL. A solution of essential oil in ethanol (1.0 mL) was combined with a solution of DPPH in ethanol (2.0 mL). The resulting mixture was agitated at a speed of 100 rpm and left undisturbed at room temperature for a duration of 30 minutes. The absorbance at a wavelength of 517 nm was determined through spectrophotometric analysis using a TU-1900 UV spectrophotometer manufactured by Persee in Beijing, China, with ethanol utilized as the reference solution. The scavenging efficacy of the compound blend against the DPPH anion radical was quantified as the SC% and determined using the equation provided by Wang L. et al. (2018):


$$SC\%=\frac{\text{A}0-\text{A}}{\text{A}0}\times 100\%$$

The initial concentration of antioxidant components in the essential oil extract of *A. sinensis* prior to its interaction with free radicals is denoted as A0, whereas the concentration of antioxidant components in the essential oil extract of *A. sinensis* following the interaction with free radicals is denoted as A.

#### 2.7.2 O_2_- free radical scavenging experiment of alcohol extract

0.1 mol/L tris solution, 1.21 g Tris mixed with distilled water to 100 mL. Tris-HCl buffer (0.05 mol/L, pH 7.4 with 1 mmol/L Na_2_EDTA), 40 mL of 0.1 mol/L Tris solution and 0.1 mol/L HCl solution and 15.2 mg Na2EDTA, mixed, diluted 80 mL. Measured with a pH meter, the pH should be 7.4. 60 mmol/L triphenol solution, take 0.1 mol/L HCl solution 20 μL, dilute to 2 mL with distilled water, to give 1 mmol/L HCl solution (pH = 2.5-3.0), and add 14.6 mg of Pyrogallol.

Determination of Pyrogallol solution (ΔA): 2950 μL of Tris-HCl buffer was taken and added to a large quartz cuvette, then added 50 μL of triphenol solution, mixed rapidly (subversion), start timing, and read every 30 seconds (A value, 325 nm) until 300 sec. (Blank reference: Tris-HCl buffer):


$$\Delta \text{A}={\text{A}}_{300\text{s}}-{\text{A}}_{30\text{s}}$$

Sample solution determination (ΔA_S_): 100 μL of sample solution was added to a large quartz cuvette, add 2850 μL of Tris-HCl buffer was taken, then added 50 μL of triphenol solution, mixed rapidly (subversive), start timing, and read every 30 seconds (A value, 325 nm) until 300 sec. (Blank reference: Tris-HCl buffer):


$$\Delta {\text{A}}_{\text{S}}={\text{A}}_{300\text{s}}-{\text{A}}_{30\text{s}}$$

Reactive oxygen species clearance (RC %):


$$RC\%=\frac{\Delta \text{A}-\Delta \text{As}}{\Delta \text{A}}\times 100\%$$

where A_30s_ is the value of readings from the spectrophotometer at 30 seconds and A_300s_ is the value of readings from the spectrophotometer at 300 seconds.

### 2.8 Data preprocessing and statistical analysis

Excel 2020 was used for test data entry, and the statistical software SPSS 23.0 (IBM, USA) was employed to conduct a one-way ANOVA analysis. All observed statistical effects demonstrated significance at a level of *P*< 0.05. One way analysis of variance (ANOVA) tests was used to analyze the yield, alcohol extract content and antioxidant activity of agarwood. The abundance accumulation diagram of endophytic fungi order and genus was made by Origin2021. Mothur version 1.2.1 was utilized for conducting rarefaction analysis to assess diversity metrics including ACE, Chao, and Shannon diversity indices (Schloss et al., 2009). The principal component analysis (PCA) figure was generated using the Vegan 2.0 package of R-forge, the community ecology software. UniFrac is used for Beta diversity analysis to contrast the results of PCA (Lozupone et al., 2011). The significance of clustering was assessed through the utilization of similarity analysis (ANOSIM) (Deng et al., 2021). Endophytic fungal symbiosis network diagram and correlation analysis were plotted using R software package.

## 3 Results

### 3.1 Agarwood yield and ethanol extract content

**Figure 1:**
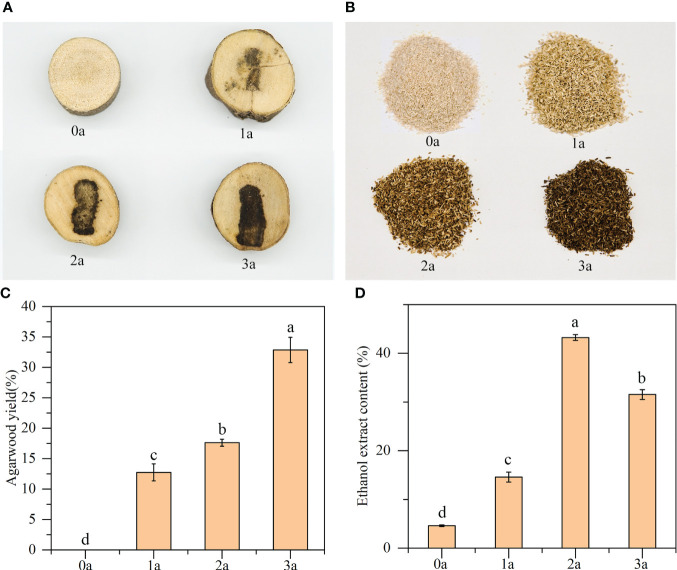
**(A–C)** The agarwood yield of Qi-Nan. **(D)** The ethanol extract content of Qi-Nan. Different letters denote significant (*P* < 0.05) differences among the treatment in a one-way ANOVA and the bar represents standards deviation (n=3). 0a, before drilling; 1a one year after treatment; 2a, two years after treatment; 3a, three years after treatment.

According to **Figures 1A, B**, as the longer the treatment period increases, the color of the agarwood becomes deeper, the area of discoloration increases, and the yield of agarwood gradually increases. Before the formation of agarwood induced by drilling, the xylem did not change color, and no agarwood was formed at that time. The agarwood yields were 12.74% of the total wood at 1a, 17.62% at 2a, and 32.86% at 3a (**Figure 1C**). Before the formation of agarwood induced by drilling, the alcohol extract content of Qi-Nan white wood was 4.60%. After 1 year, the content of alcohol extract in Qi-Nan agarwood increased to 14.58%. At 2 years, the content of alcohol extract in Qi-Nan agarwood further increased to 43.24%. Finally, at 3 years, the content of alcohol extract in Qi-Nan agarwood decreased slightly to 31.54% (**Figure 1D**).

### 3.2 Substance distribution and transformation during agarwood formation

**Figure 2:**
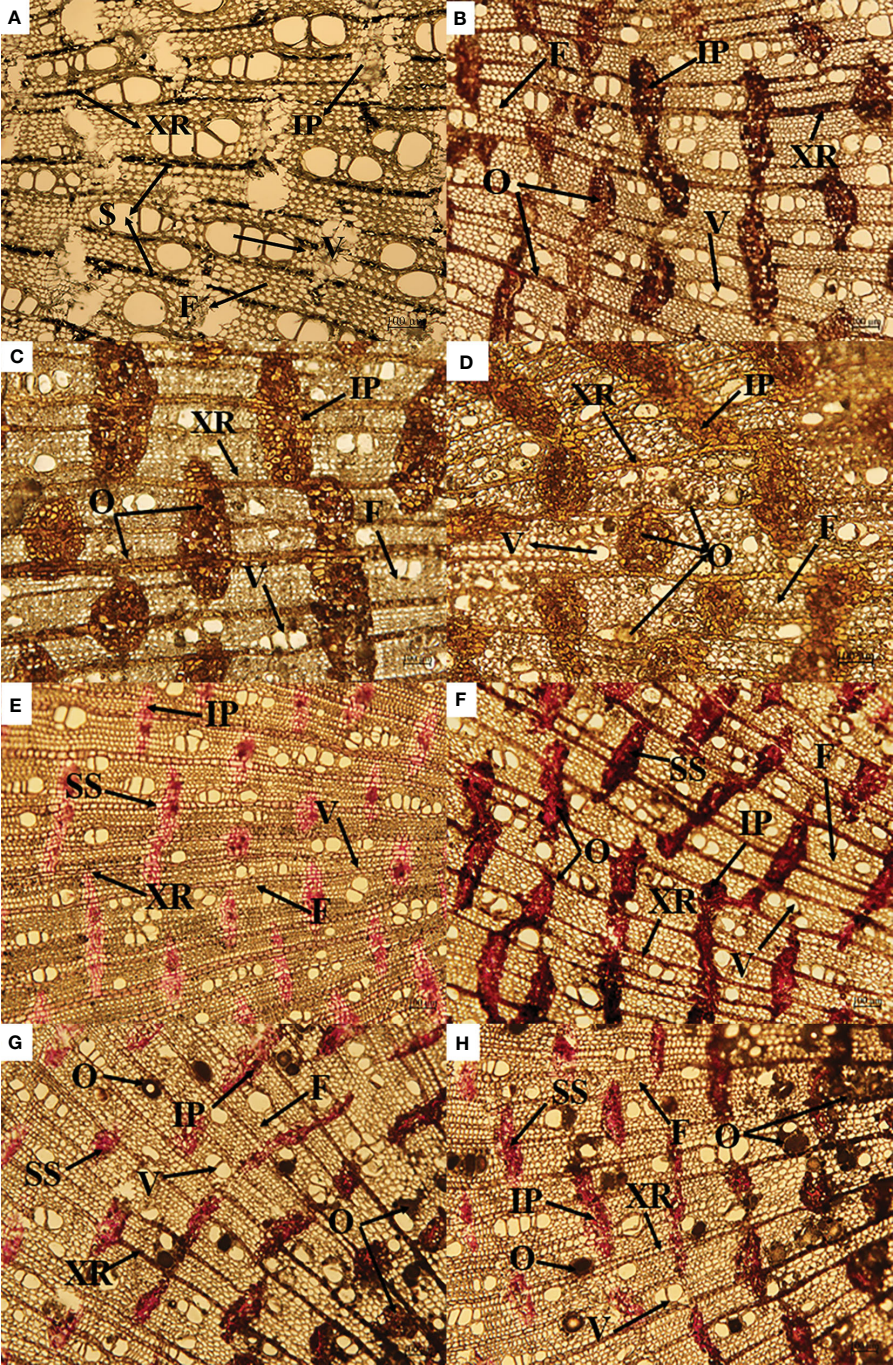
Changes in starch and agarwood formation over time on cross-section **(A–D)**. Changes is soluble sugar and agarwood formation over time on cross-section **(E–H)**. IP, interxylary phloem; S, starch; F, xylem fiber; V, xylem vessel; XR, xylem ray; O, oil; SS, soluble sugar.

As shown in **Figure 2**, most of the starch existed in the xylem ray cells, a small amount existed in the interxylary phloem (**Figure 2A**), most of the soluble sugar existed in the interxylary phloem, and a small amount existed in the xylem ray cells (**Figure 2E**).

After the agarwood was formed, the starch granules disappeared, and the oily substances filled the interxylary phloem and xylem rays. With increasing induction time, the color of the oily substances in the interxylary phloem and xylem rays became lighter and the distribution was more uniform. After three years of induction, the xylem vessels were also filled with oily substances, and the oily substances were evenly distributed in the xylem (**Figures 2A–D**).

The soluble sugar content of *A. sinensis* first increased and then decreased after agarwood formation (**Figures 2E–H**). With increasing induction time, the soluble sugars in xylem rays and interxylary phloem disappeared and were replaced by oily substances (**Figures 2G, H**).

### 3.3 Chemical composition analysis of Qi-Nan agarwood

**Figure 3:**
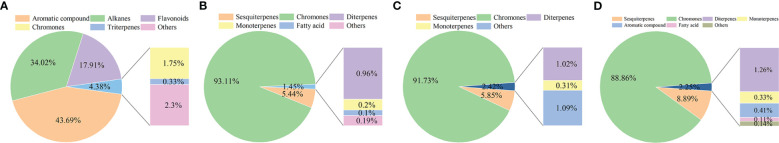
The changes of chemical composition of Qi-Nan before and after drilling for 1, 2, 3 years. **(A)** The component composition of white wood of Qi-Nan before drilling. **(B)** The component composition of Qi-Nan agarwood after 1 year drilling. **(C)** The component composition of Qi-nan agarwood after 2 years of drilling. **(D)** The component composition of Qi-Nan agarwood after 3 year of drilling.

A total of 72 compounds were found in Qi-Nan white wood and Qi-Nan agarwood, including chromones, sesquiterpenes, monoterpenes, diterpenes, triterpenes, fatty acids, alkanes, flavonoids and aromatic compounds (**Supplementary Table S1**, **Figure 3**). A total of 22 compounds were identified in Qi-Nan white wood, primarily consisting of aromatic compounds, alkanes, and flavonoids. After the formation of Qi-Nan agarwood, the content of these substances decreases rapidly. There were 37 compounds in the agarwood induced after 1 year, 32 compounds in the agarwood induced after 2 years, and 36 compounds in the agarwood induced after 3 years. Chromones and sesquiterpenes were identified as the main components of Qi-Nan agarwood produced at different ages (**Figure 3**). The relative contents of chromones and sesquiterpenes were accounted for 93.11% and 5.44% of the total primary components in Qi-Nan agarwood induced after 1 year, 91.73% and 5.85% in agarwood induced after 2 years and 88.86% and 8.89% in agarwood induced after 3 years. With the prolongation of induction time, the relative content of chromones in agarwood decreased (**Figure 3**), while the relative content of sesquiterpenes in agarwood increased (**Supplementary Table S1**).

Chromones found in Qi-Nan agarwood aged 1 year, 2 years, and 3 years were 9, 6, and 9 kinds, respectively. 2-(2-Phenethyl) chromone, 2-[2-(4-Methoxyphenyl)ethyl] chromone, and 2-[2-(3-Methoxy-4-hydroxyphenyl)ethyl] chromone were the most abundant chromones, with an average relative content of 24.97%, 37.53%, and 20.34% (**Supplementary Table S1**). Only 2- [2- (4-Methoxyphenyl) ethyl] chromone was found in Qi-Nan white wood, and the relative content was only 1.75%. In comparison to 1 year and 2 years Qi-Nan agarwood, the relative content of 2- (2-phenylethyl) chromone was significantly higher in 3 years Qi-Nan agarwood, while the relative content of 2-[2-(3-Methoxy-4-hydroxyphenyl) ethyl] chromone was significantly lower in 3-year Qi-Nan agarwood (**Supplementary Table S1**).

Sesquiterpenes were found in Qi-Nan agarwood aged 1, 2 and 3 years, with 15, 19, and 23 types respectively, each having relative contents greater than 0.1%. However, no sesquiterpenoids were identified in Qi-Nan white wood. With an increase in induction time, the type and content of sesquiterpenes steadily increased (**Supplementary Table S1**).

### 3.4 Endogenous fungal variation characteristics of Qi-Nan

#### 3.4.1 Sequence characteristics and Qi-Nan endogenous fungal community diversity

A total of 12 samples (0a, 1a, 2a and 3a after induction with 3 replicates) were taken and sequenced and 662692 endophytic fungal sequences were obtained. After eliminating 88055 sequences that were identified as low-quality, non-fungal, potential chimeras and singletons, the residual non-chimeric fungal internal transcribed spacer 2 (ITS2) sequences, totaling 574,637, were grouped into 1,797 non-singleton operational taxonomic units (OTUs) at a 97% sequence similarity threshold. Among the 1797 OTUs, 1637 were identified as fungal species (**Supplementary Table S2**). The Venn diagram illustrated that 587 core OTUs were common among all the samples (**Supplementary Figure S1**).

**Figure 4:**
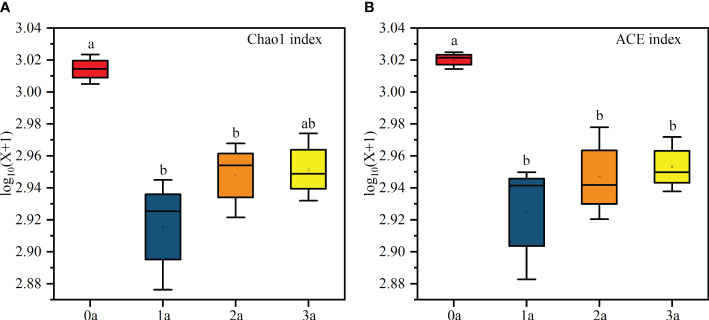
Chao 1 **(A)** and ACE **(B)** index of Qi-Nan endophytic fungi communities with different induction time (n=3). 0a, before drilling; 1a; one year after drilling; 2a, two years after drilling; 3a, three years after drilling. Different letters denote significant (*P* < 0.05) differences among the treatment in a one-way ANOVA and the bar represents standard deviation (n=3).

Alpha diversity analysis showed that the Chao1 index and ACE index were the highest when the tree was not induced and the lowest after one year of induction, and then increased with the induction time (**Figure 4**). In addition, the Reads, Richness, Shannon, Simpson and Pielou indices had no significant differences (**Supplementary Table S3**). The diversity of endophytic fungi decreased first and then increased slowly after induction of agarwood formation. Coverage was above 97% for each sample, suggesting that the sequencing depth effectively reflected the actual richness of the Qi-Nan endophytic fungal communities (**Supplementary Table S2**).

#### 3.4.2 Qi-Nan endophytic fungal community structure

**Figure 5:**
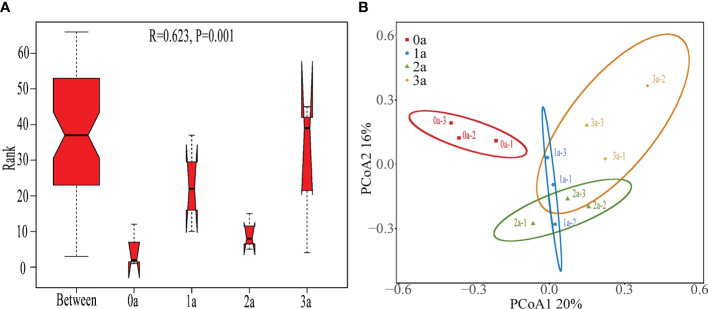
ANOSIM **(A)** and PCoA **(B)** of Qi-Nan endophytic fungi in different induction time. 0a, before drilling; 1a one year after drilling; 2a, two years after drilling; 3a, three years after drilling.

The ANOSIM findings (R = 0.623, *P*< 0.001) demonstrated a statistically significant variation in the Qi-Nan endophytic fungal communities due to the duration of induction (**Figure 5A**). It was obvious from the PCoA that there were significantly different clusters before and after the induction of agarwood formation (**Figure 5B**).

#### 3.4.3 Qi-Nan endophytic fungal community composition and evolutionary development

For the Qi-Nan endophytic fungal communities, 574637 high-quality sequences assigned to 4 phyla, 73 orders and 448genera were found and provided a specific analysis by order and genus (relative abundance exceeded 1%).

**Figure 6:**
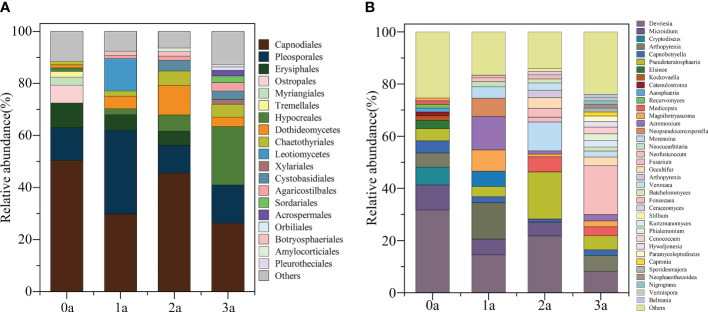
The classification of Qi-Nan endophytic fungi communities at order levels **(A)** and genus levels **(B)** (relative abundance exceeded 1%) under different induction time and the relative abundance of each order and genus. 0a, before drilling; 1a; one year after drilling; 2a, two years after drilling; 3a, three years after drilling.

After one year of induction of agarwood, there was no increase in the order level of Qi-Nan endophytic fungi to 9 orders, and after two years of induction, there were 10 orders, and after three years of induction of agarwood, there were 13 orders. With induction time, the endophytic fungi in Qi-Nan stems increased at the order level (**Figure 6A**). At different induction times, the dominant orders (relative abundance exceeded 10%) of Qi-Nan endophytic fungi were also different. Before induction of agarwood formation, the dominant endophytic orders were *Capnodiales* (50.47 ± 12.77%) and *Pleosporales* (12.49 ± 3.97%); one year after induction, they were *Pleosporales* (32.13 ± 2.08%), *Capnodiales* (29.84 ± 17.81%) and *Leotiomycetes* (12.50 ± 6.83%); two years after induction, they were *Capnodiales* (45.59 ± 15.10%), *Dothideomycetes* (11.33 ± 3.77%) and *Pleosporales* (10.67 ± 3.76%); and three years after induction, they were *Capnodiales* (26.29 ± 14.71%), *Hypocreales* (22.43 ± 8.74%) and *Pleosporales* (14.70 ± 4.15%) (**Figure 6A**; **Supplementary Table S4**). *Capnodiales* and *Pleosporales* are the common dominant endophytic fungi of Qi-Nan in different induction periods.

**Figure 7:**
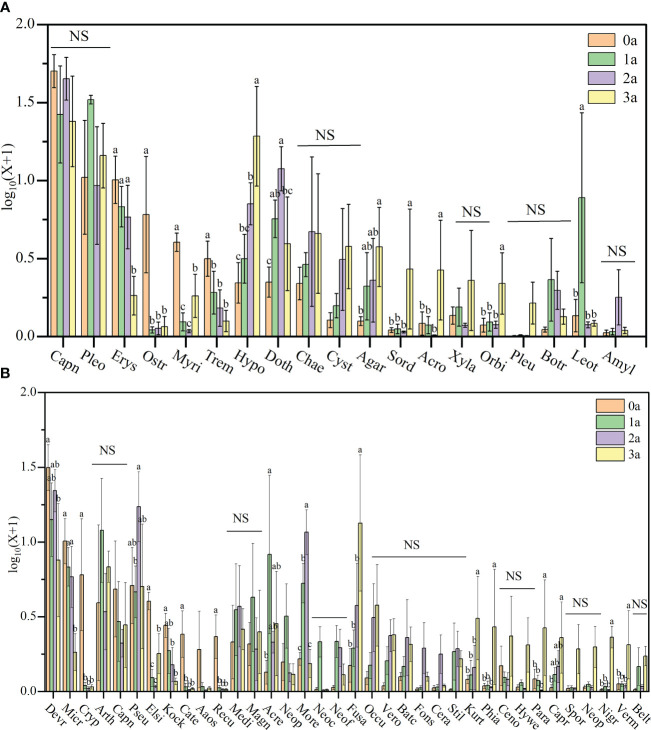
The relative abundance of Qi-Nan endophytic fungi communities at order levels **(A)** and genus levels **(B)** under different induction time. Different letters indicate significant differences under different induction time (*P* < 0.05), “NS” shows no significant difference. 0a, before drilling; 1a; one year after drilling; 2a, two years after drilling; 3a, three years after drilling. The abscissa in the figure corresponds to the order and genus of endophytic fungi in **Figure 6**.

The genus number of Qi-Nan endophytic fungi increased increasing induction time of agarwood formation. There were 13 genera in Qi-Nan endophytic fungi before the induction of agarwood formation, and 13 genera one year after induction; 17 genera two years after induction, and 22 genera three years after induction. With induction time, the endophytic fungi in Qi-Nan stems increased at the genus level (**Figure 6B**). At different induction times, the dominant genera (relative abundance exceeded 10%) of Qi-Nan endophytic fungi were also different. Before induction of agarwood formation, the dominant endophytic genera were *Devriesia* (31.75 ± 5.24%); one year after induction, they were *Devriesia* (14.48 ± 7.12%), *Arthopyrenia* (14.02 ± 2.88%) and Acremonium (12.86 ± 6.77%); two years after induction, they were *Devriesia* (21.78 ± 6.71%), *Pseudoteratosphaeria* (18.11 ± 3.22%) and *Morenoina* (11.08 ± 3.88%); and three years after induction, they were *Fusarium* (26.29 18.71 ± 1.19%) (**Figure 7B**; **Supplementary Table S4**). Only *Devriesia* was found to be the common dominant endophytic fungus before induction, one year after induction and two years after induction of Qi-Nan.

**Figure 8:**
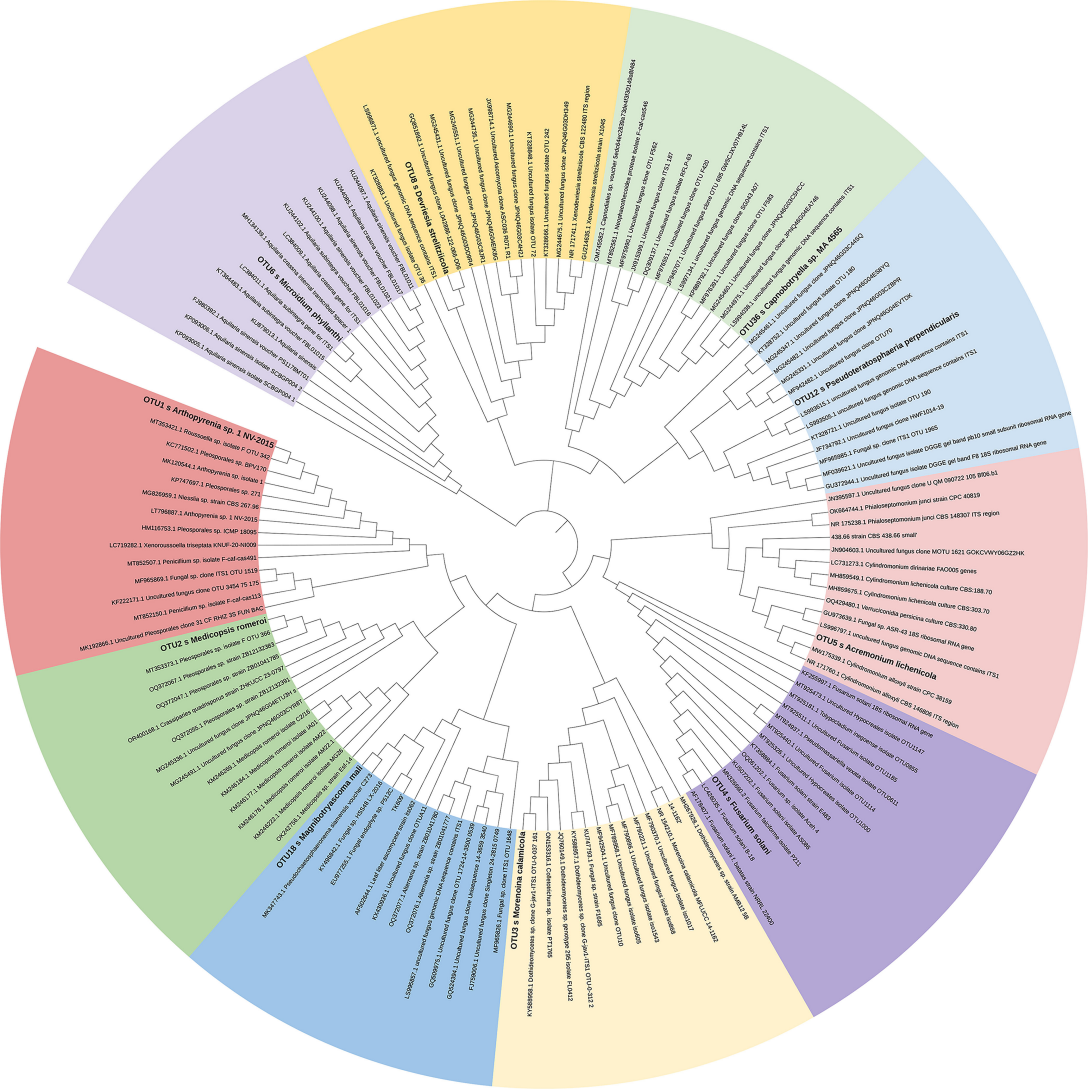
The phylogentic relationship of the top ten genera of endophytic fungi in Qi-Nan agarwood.

After constructing a phylogenetic tree of the top ten endophytic fungi of Qi-Nan (**Figure 8**; **Supplementary Table S5**), it can be found that 10 endophytic fungi are on three large branches, *Microidium* is a single large branch and is unique to the *A. sinensis* tree. *Devriesia*, *Capnobotryella* and *Pseudoteratosphaeria* are in a large branch, but *Capnobotryella* and *Pseudoteratosphaeria* have a closer genetic relationship and most of the related endophytic fungi are unculturable fungi. *Acremonium*, *Fusarium*, *Morenoina*, *Magnibotryascoma*, *Medicopsis* and *Arthopyrenia* are in a large branch, and *Medicopsis* and *Arthopyrenia* are closely related.

Regarding the Qi-Nan endophytic fungal order, *Erysiphales* (0.88 - 9.54%), *Ostropales* (0.11 - 6.84%), *Myriangiales* (0.09 - 3.06%), *Tremellales* (0.27 - 2.23%), *Hypocreales* (1.28-22.43%), Dothideomycetes (1.27 - 11.33%), *Agaricostilbales* (0.26 - 3.25%), *Sordariales* (0.12-2.58%), *Acrospermales* (0.02 - 2.13%), *Orbiliales* (0.19 - 1.34%), and *Leotiomycetes* (0.19 - 12.50%) showed significant differences during different induction periods of Qi-Nan (**Figure 7A**). Drill-to-induction time significantly reduced the abundance of *Erysiphales*, *Ostropales*, *Myriangiales*, *Tremellales* and significantly increased the abundance of *Hypocreales*, *Agaricostilbales*, *Sordariales*, *Acrospermales*, *Orbiliales*. The abundance of *Dothideomycetes* and *Leotiomycetes* first increased and then decreased with increasing induction time. *Dothideomycetes* had the lowest abundance before drilling to induce agarwood formation and the greatest abundance at two years of drill to induction agarwood formation. *Leotiomycetes* had the lowest abundance before drilling to induce agarwood formation and the greatest abundance after three years of drilling to induce agarwood formation. (**Figure 7A**).

Regarding the Qi-Nan endophytic fungal genera, *Devriesia* (8.26-31.75%), *Microidium* (0.88-9.53%), *Cryptodiscus* (0.05-6.83%), *Pseudoteratosphaeria* (3.89-18.11%), *Elsinoe* (0.08-3.05%), *Kockovaella* (0.18-1.80%), *Catenulostroma*(0.03-1.53%), *Recurvomyces* (0.03-1.42%), *Acremonium* (0.36-12.86%), *Morenoina* (0.56-11.08%), *Fusarium* (0.53-18.71%), *Kurtzmanomyces* (0.21-2.59%), *Phialemonium* (0.06-2.58%), *Paramycoleptodiscus* (0.02-2.13%), *Capronia* (0.06-1.51%), *Nigrograna* (0.02-1.33%) and *Vermispora* (0.08-1.25%) showed significant differences during different induction periods of Qi-Nan (**Figure 7B**. Drill to induce agarwood formation significantly reduced the abundance of *Devriesia*, *Microidium*, *Cryptodiscus*, *Elsinoe*, *Kockovaella*, *Catenulostroma*, *Recurvomyces*, and significantly increased the abundance of *Fusarium*, *Kurtzmanomyces*, *Phialemonium*, *Paramycoleptodiscus*, *Capronia*, *Nigrograna*, *Vermispora*. The abundance of *Pseudoteratosphaeria*, *Acremonium* and *Morenoina* first increased and then decreased with increasing induction time. *Pseudoteratosphaeria* had the lowest abundance after one year of drilling to induce agarwood formation and the greatest abundance at two years of drilling to induce agarwood formation. *Acremonium* had the lowest abundance before drilling and the greatest abundance at one year after drilling, *Morenoina* had the lowest abundance before drilling and the greatest abundance at two years after drilling (**Figure 7B**).

#### 3.4.4 Co-Occurrence network of Qi-Nan endophytic fungi community

**Figure 9:**
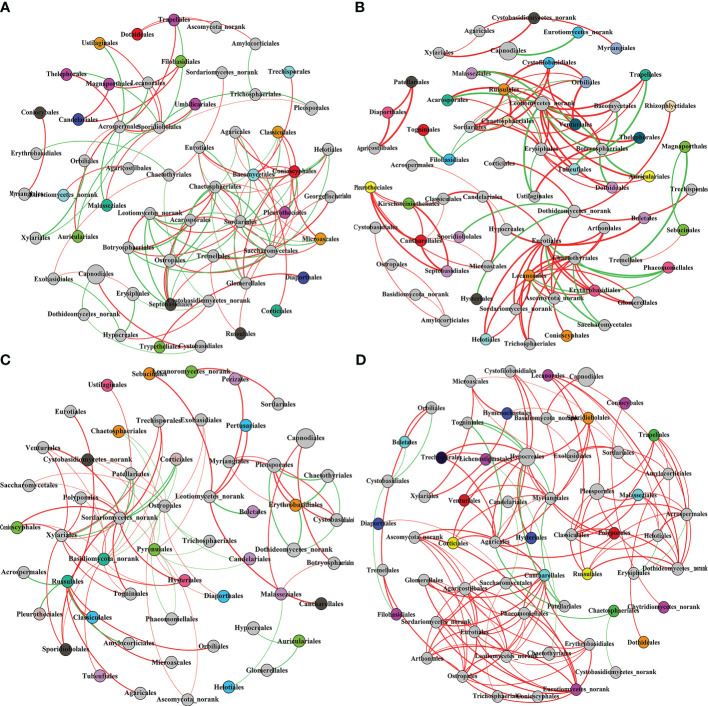
The co-occurrence network of Qi-Nan endophytic fungi communities at the order level in different induction time. Each node size is based on their relative abundance. The red and green edges represented the positive and negative correlation between Qi-Nan endophytic coefficient. **(A)** Before drilling; **(B)** one year after drilling; **(C)** two years after drilling; **(D)** three years after drilling.

Co-Occurrence network is a Pearson’s correlation analysis of the Qi-Nan endophytic fungal communities. Networks were formed using OTUs of the Qi-Nan endophytic fungi order. These networks comprise 111, 111, 81, and 134 edges linking 57, 65, 55, and 59 nodes, respectively (**Figure 9**, **Table 1**). The number of nodes was the most at one year of induction, and the edge was the most at three years of induction. With increasing induction time, the positive correlation relationship of endophytic fungi was strengthened (54.95-89.00%), and the negative correlation relationship was weakened (45.95-11%) (**Table 1**). The modularity, average path length and network diameter reached a maximum at two years of induction, and the clustering coefficient and graph density reached a maximum at three years of induction. Before induction of agarwood formation, the highly connected nodes of endophytic fungi were *Sordariales*, *Acrospermales* and *Helotiales*; one years after induction, the highly connected nodes of endophytic fungi were *Erysiphales*, *Chaetothyriales*, *Leotiomycetes*, *Amylocorticiales*; two years after induction, the highly connected nodes of endophytic fungi were *Botryosphaeriales*, *Amylocorticiales*, *Agaricales*; and three years after induction, the highly connected nodes of endophytic fungi were *Hypocreales*, *Leotiomycetes* and *Agaricostilbales*.

**Table 1:** Qi-Nan endophytic fungal community co-occurrence network for different induction times.

Topological properties	0a	1a	2a	3a
Nodes	57	65	55	59
Edges	111	111	81	134
positive (red)	54.95%	76.58%	66.67%	89.00%
negative (green)	45.95%	23.42%	33.33%	11.00%
Clustering coefficient	0.679	0.494	0.403	0.737
Modularity	0.59	0.766	0.765	0.756
Average path length	1.873	2.361	1.644	1.439
Network diameter	5	6	3	4
Graph density	0.07	0.053	0.055	0.078
Average degree	3.895	3.415	2.945	4.542

0a, before drilling;1a, one year after drilling; 2a, two years after drilling; 3a, three years after drilling.

### 3.5 Antioxidant activity tests of Qi-Nan

**Figure 10:**
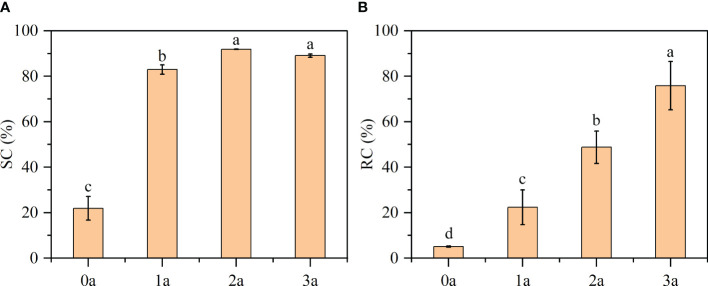
Results of **(A)** DPPH free radical scavenging activity (SC) and **(B)** reactive oxygen species clearance (RC). Different letters denote significant (*P* < 0.05) differences among the treatment in a one-way ANOVA and the bar represents standard deviation (n = 3).

DPPH, a nitrogen-centered free radical, exhibits remarkable stability and possesses the ability to scavenge other free radicals. It demonstrates a pronounced absorption peak at 517 nm. It can be used as a good oxidation resistance detection reagent, when the freshly prepared DPPH reacts with an antioxidant solution, while purple will become pale yellow or colorless (Wang et al., 2018). From **Figure 10A**, the order of decrease in the DPPH scavenging effect was as follow: 2a (91.88%) > 3a (89.06%) > 1a (82.96%) > 0a (21.90%) (before drilling and 1a, 2a, 3a after induction). Qi-Nan had the lowest scavenging activity (SC%) before drilling. With increasing time, the scavenging activity was strengthened, was the strongest at 2 years, and then decreased in the third year. According to the existing literature, a substance is deemed to possess antioxidant capacity if its SC_50_ value, which represents the concentration of essential oil at which the SC% reaches 50%, is less than 10 mg/ml (Wang L. et al., 2018). In this experiment, the one-year formation of Qi-Nan was 7 mg/ml, so we know that Qi-Nan has strong scavenging activity.

Reactive oxygen species are present in both the human body and the plant body and can be used to test whether an extract has antioxidant activity or the strength of the activity (Li, 2012). From **Figure 10B**, with increasing induction time, the reactive oxygen species clearance (RC %) of Qi-Nan increased, and was the strongest at three years, reaching 75.84%. Followed by 2a (48.77%), 1a (22.37%), 0a (5.09%).

### 3.6 Correlation between components and endophytic fungi of Qi-Nan agarwood

**Figure 11:**
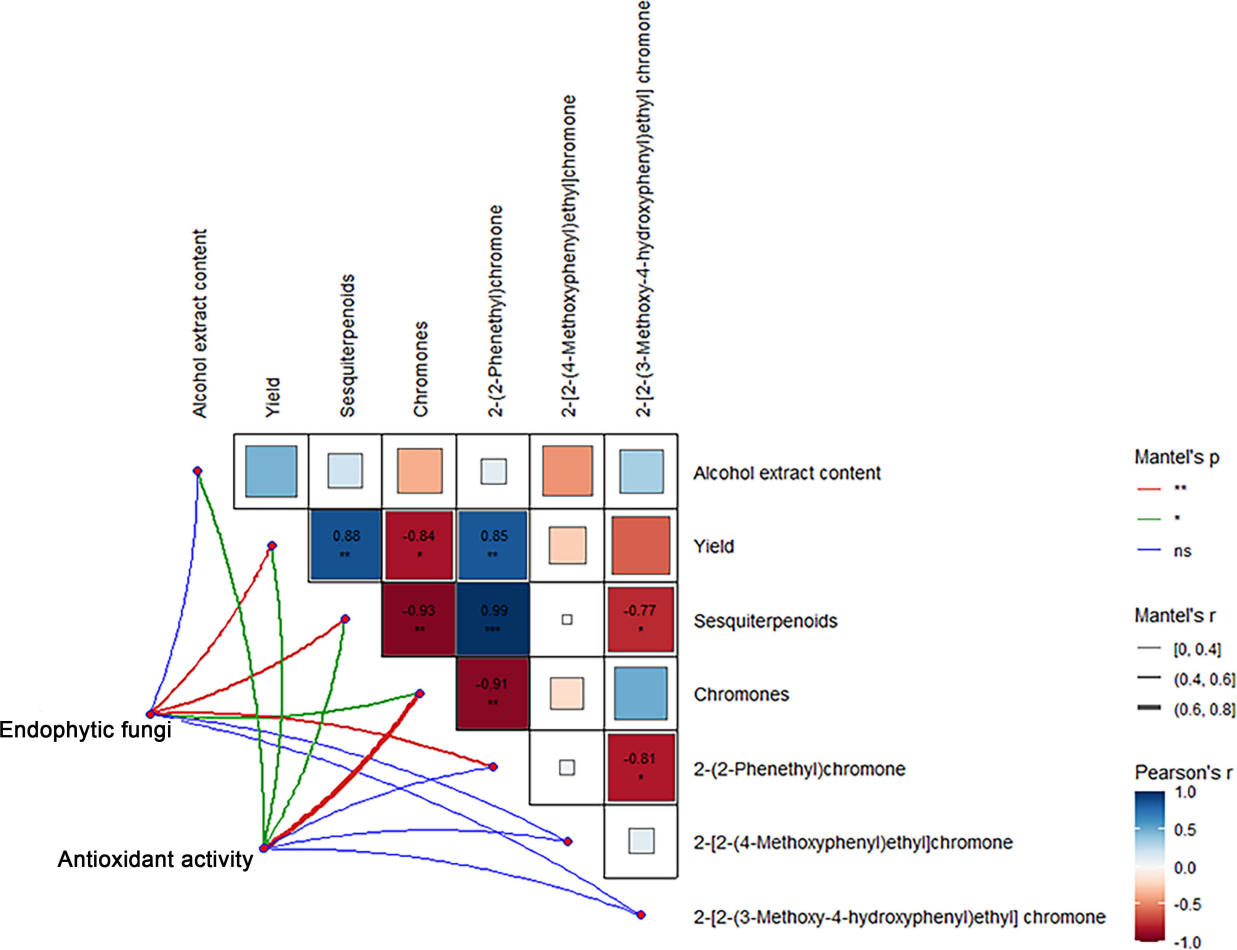
Correlation between components, active free radical scavenging ability and endophytic fungi of Qi-Nan agarwood. ***, P<0.001, indicates strong correlation.

Correlation analysis showed that the sesquiterpene content and 2-(2-phenylethyl) chromone content in Qi-Nan agarwood were significantly positively correlated with the agarwood yield, and the chromones content exhibited a notable negative correlation with both the yield and the sesquiterpenes content. The 2-(2-phenylethyl) chromone content exhibited a notable positive association with sesquiterpene content, while displaying a negative correlation with chromone content. The 2-[2-(3-Methoxy-4-hydroxyphenyl) ethyl] chromone content was significantly negatively correlated with the content of sesquiterpenes and 2-(2-phenylethyl) chromone (**Figure 11**).

From **Figure 11**, the endophytic fungi of Qi-Nan agarwood were significantly positively correlated with the yield, sesquiterpene content, chromone content and 2-(2-phenylethyl) chromone content. The active free radical scavenging ability of Qi-Nan agarwood was significantly positively correlated with the content of alcohol extract, the yield of Qi-Nan agarwood, and the content of sesquiterpenes and chromones.

## 4 Discussion

### 4.1 Material metabolism and tissue structure basis of Qi-Nan agarwood formation

In comparison to trees from ordinary seed seedlings following artificial induction, Qi-Nan clones are a high-quality cultivar that is reproduced through the process of grafting onto ordinary seed seedlings of *A. sinensis* and can more easily yield higher grade agarwood (Wang et al., 2020; Yu et al., 2021). According to recent study, the species of the Qi-Nan clone is *A. sinensis* (Kang et al., 2022); nevertheless, when exposed to wounding stress, the Qi-Nan germplasm elicited distinct biological responses than ordinary *A. sinensis* (Lv et al., 2022).

Agarwood is a resinous compound that is synthesized by plants in response to physical injuries and pathogen invasions (Karlinasari et al., 2015). When the *A. sinensis* tree experiences external stress, it utilizes non-structural carbohydrates as metabolic substrates to form a glue between secondary metabolites and xylem, thereby preventing further damage (Li et al., 2015; Adams et al., 2016; Kuo et al., 2020). Consumption of starch to form agarwood is often divided into two stages, and two distinct physiological alterations can be observed during the development of agarwood. The first change observed after tree injury is a decrease and disappearance of starch in parenchyma cells. The second change is the significant saponification in parenchymal tissue cells, resulting in the appearance of brown droplets (Kono et al., 2019; Li et al., 2022). This study showed that the development of Qi-Nan agarwood consumed starch and soluble sugar in parenchyma cells, and the agarwood formed was filled in the xylem. Starch disappeared quickly after agarwood formation, and soluble sugar decreased with increasing agarwood formation time. When the agarwood is completely formed, the soluble sugar is consumed completely.

Several studies have indicated that non-structural carbohydrates were found in high quantities in the interxylary phloem and xylem ray cells. Additionally, the brown agarwood resin was primarily concentrated in the interxylary phloem and xylem ray cells following agarwood formation, with a lesser presence in the xylem vessels and fibers (Faizal et al., 2016; Liu et al., 2019). Moreover, during the initial stages of agarwood formation, the observation revealed a significant abundance of mitochondria and plastids within the parenchyma cells. Researchers hypothesize that these organelles play a crucial role in providing energy and serving as sites for the synthesis of agarwood resin (Liu et al., 2019). Meanwhile, three types of osmiophilic substances, which are thought to have a strong connection to the development of agarwood, were observed in these parenchyma cells. Research carried out by Liu et al. (2019) has shown that the development of agarwood is characterized by the occurrence of apoptosis in various parenchyma cells, including axial parenchyma, interxylary phloem and xylem rays. The conversion of starch grains into soluble sugar, the synthesis of sesquiterpenes and the synthesis of other unique components of agarwood can be observed in different parenchyma cells. The main process of material change took place predominantly in the interxylary phloem, establishing a structural and material basis for the development of agarwood (Liu Y. et al., 2022). In the present study, the formation of Qi-Nan agarwood was observed in the interxylary phloem and xylem ray cells. Therefore, there is compelling evidence to suggest that the interxylary phloem and xylem ray cells play a crucial role in the formation and storage of Qi-Nan agarwood, while the xylem active parenchyma cells serve as the fundamental tissue for the formation of Qi-Nan agarwood.

### 4.2 Changes in the chemical composition of Qi-Nan agarwood

According to studies, agarwood contains a wide range of substances, including terpenoids, flavonoids, lignans, and steroids (Chen H. Q. et al., 2012; Li et al., 2015; Li et al., 2020). Moreover, several studies have documented that the main compounds of agarwood are fatty alkanes, whereas the primary constituents of agarwood consist of aromatic and sesquiterpenoids compounds (Wang M. R. et al., 2018; Yang et al., 2018). 2-(2-phenylethyl) chromones and sesquiterpenes have been identified as the main characteristic chemical ingredients of agarwood and exhibited various of biological (Liao et al., 2018; Yang et al., 2019). We have discovered that the main components of Qi-Nan agarwood are sesquiterpenes and 2-(2-phenylethyl) chromones, which aligns with findings from previous research.

Studies have shown that when the *Aquilaria* tree is invaded by fungi, the starch granules in the active cells of the xylem are converted into non-starch polysaccharides and phenols. These intermediates undergo a sequence of chemical reactions to be transformed into chromones and sesquiterpenes (Cui et al., 2013). Fungal infection typically results in elevated production of free fatty acids, which leads to oxidative bursts, resulting in the production of oxygenated compounds, including jasmonate (Sen et al., 2017), and found that jasmonic acid (JA) can be synthesized by endophytic strains in *A. sinensis* (Chen et al., 2017). It is widely recognized that JA plays a significant role as a key signaling molecule in triggering the biosynthesis of sesquiterpenes and chromone derivatives in *A. sinensis* (Faizal et al., 2021). Interestingly, some endophytic fungi in *A. sinensis* can also synthesis sesquiterpenes, including *Acremonium* sp., *Collectotrichum* sp., *Fimetariella rabenhorstii*, *Nigrospora oryzae*, and *Nodulisporium* sp. accountable for the synthesis of sesquiterpenes (Monggoot et al., 2017; Tibpromma et al., 2021). Therefore, when the diversity of endophytic fungi changes, the components of agarwood will also change. Sesquiterpenes and chromones accumulate rapidly in the early stage of agarwood formation to prevent further damage by fungi (Okudera and Ito, 2009). In addition, the age of the tree, the external environment, and the formation time of the induced agarwood may also affect the changes in the composition of the agarwood (Zhang et al., 2009). The materials used in this study were the same age of the same origin. Therefore, the biggest factor affecting the composition of the agarwood is the induction of the formation time of the agarwood. Zhang (2013) conducted an analyzed on ordinary agarwood samples that were induced on the 15th, 30th, and 60th days by using GC-MS, and the results showed the detection of sesquiterpenes after 60 days. Furthermore, the proportion of aromatic and sesquiterpenes compounds showed an increase with prolonged time, which aligns with our findings. Wu et al. (2020) found that in ordinary *A. sinensis* trees, the relative content of chromones increased with induction time within 6 months. In contrast, in Qi-Nan agarwood, we found that the proportion of chromones decreased as the induction time increased, though it was still the highest content component, which may be related to the mutual transformation between substances and the time of inducing the formation of agarwood. Liao et al. (2018) found that chromones can form other types of chromones by a higher degree of hydroxylation or methoxylation, and these chromones may not be detected. Ma et al. (2021) found that during the induction of *A. sinensis*, the content of chromone continued to increase from 6 to 12 months and the content of chromone decreased when the induction time exceeded 12 months. Qi et al. (2005) used *Aspergillus* flavus extract to culture *A. sinensis* cell suspension and found that the content of chromone in cells increased rapidly in a short time, and the content of chromone in cells decreased after a period of culture time. During the continuous defense process, certain defensive compounds like chromone were successful in decreasing the abundance of fungi, resulting in a diminished defense response in *A. sinensis*, and the synthesis rate of chromone also continued to decrease (Mohamed, 2016). This may be the main reason for the decrease of chromone content after long-term induction.

### 4.3 Endophytic fungi promoting the formation of Qi-Nan agarwood

As the fungi infects the *A. sinensis* trees, self-immune function enables trees to react to pathogens by stimulating the synthesis of defensive metabolites that coordinate immune reactions (Couto and Zipfel, 2016; Kourelis and Hoorn, 2018; Xin et al., 2018), and the tree secretes a substantial amount of resin containing volatile organic compounds, which assist in inhibiting or slowing down the proliferation of the fungus (Sangareswari et al., 2016). Research has indicated that during the initial phases of fungal infection, the enzymatic degradation byproducts of the cuticle serve as the intrinsic cellular signal recognized by the plant, acting as the initial elicitor of a defensive reaction (Serrano et al., 2014). *A. sinensis* trees often form a glue of wood fiber and resin to effectively segment damage and infection (Hasibuan et al., 2013). Therefore, the diversity of endophytic fungi decreased after agarwood formation in Qi-Nan agarwood. Under normal conditions, the xylem is infected by fungi after injury, and rotten wood is formed during damage and pathogen-plant interaction (Blanchette, 1992), so the diversity of endophytic fungi increased slowly two years after the formation of Qi-Nan agarwood, probably because the wound at the perforation was infected and slightly rotted. Different from our results, Liu J. et al. (2022) found that the diversity of endophytic fungi in resin wood is much richer than that in healthy wood of *Aquilaria*. It may be because the previous studies were ordinary *A. sinensis*, and the yield of agarwood formed by ordinary *A. sinensis* was often less than 10%, and most of the discoloration range was rotten wood (Zhang et al., 2022). Therefore, the diversity of endophytic fungi increased in the third year. When Qi-Nan agarwood formed, the yield could easily exceed 10%, and the rotten part was very few. Moreover, Qi-Nan agarwood had high chromone and terpenes content and strong antibacterial property (Zulak and Bohlmann, 2010; Naef, 2011), so the diversity of endophytic fungi decreased during the formation of Qi-Nan agarwood.

The endophytic fungi and *A. sinensis* maintained a symbiotic relationship before the formation of agarwood, which would not cause any harm. When the development of agarwood is induced, the tree body of *A. sinensis* is damaged, the balance of microecosystem is broken, and some endophytic fungi will grow rapidly, causing the defense response of agarwood tree body (Liu Y. et al., 2022). The active cells in the xylem use starch and soluble sugar as metabolic substrates to produce a large number of secondary metabolites, including bioactive substances such as sesquiterpenes and chromones (Li et al., 2022). Agarwood is formed and filled in the xylem. At the same time, the active cells in the xylem filling area die in large numbers, and starch and soluble sugar are also consumed in large quantities. Due to the large increase of bioactive substances and the large reduction of metabolic substrates of endophytic fungi, a significant quantity of endophytic fungi in the agarwood area will die and decompose, resulting in a decrease in the diversity of endophytic fungi after agarwood formation (Li et al., 2022).

It was found that a total of 42 fungal families and 67 fungal genera were isolated and identified across eight agarwood-producing taxa, with the majority (82.8%) falling under *Ascomycota* (Li et al., 2023). Most of the endophytic fungi in *Ascomycota* belong to *Dothideomycetes*, while the others to *Sordariomycetes* or *Eurotiomycetes* (Chen et al., 2017). Different from the results of previous studies, in our study, it was found that the endophytic fungi of *A. sinensis* were mainly identified as 4 phyla, 73 orders, 448 genera, and most of them belonged to *Ascomycota* and *Basidiomycota*. This may be due to different research methods, not all endophytes may have been isolated since some may not grow under laboratory conditions, some may grow too slowly to be found (Hyde and Soytong, 2008). In addition, the separation method also determines the diversity of the endophytic fungi to a large extent (Das et al., 2007). We use the metagenomic approach to largely overcome the limitations of the culture-based approach.

Many endophytic fungi have been isolated and identified from agarwood, such as *Alternaria*, *Botryosphaeria*, *Cephalosporium*, *Cladophialophora*, *Cladosporium*, *Curvularia*, *Epicoccum*, *Fusarium*, *Geotrichum*, *Glomerularia*, *Gonytrichum*, *Guignardia*, *Hypocrea*, *Lasidiplodia*, *Monilia*, *Mortierella*, *Mycelia sterilia*, *Ovulariopsis*, *Penicillium*, *Phaeoacremonium*, *Pleospora*, *Preussia, Rhinocladiellas* and *Trichoderma* (Promputtha et al., 2004; Gong and Guo, 2009; Tian et al., 2013; Liu J. et al., 2022), and some of them are consistent with this study, but some are different. Studies have shown that endophytic fungi can improve the ecological adaptability of the host, and different endophytic fungi can induce different compounds to improve the resistance of the host to different environmental stresses. Similarly, the endophytic fungi in the host will be different in different environments (Monggoot et al., 2017). Therefore, endophytic fungi in *Aquilaria* plants are affected by their geographical location, climate, light, soil moisture and nutrients. Endophytic fungi may be different under different environmental conditions (Chen et al., 2019), while different strains may also cause different endophytic fungi, which may explain why our results are different from others. In addition, the study found that *Acremonium* was the predominant genus in the undamaged xylem of agarwood, while *Penicillium* was the prevailing genus in the resin-forming region (Li et al., 2023). *Colletotrichum* was as the generally dominant species and *Fusarium* was as the specifically dominant species in agarwood (Tian et al., 2013). We found that the dominant endophytic fungi of Qi-Nan changed with the induction times, *Devriesia* was the dominant fungi genus before agarwood formation, *Devriesia*, *Arthopyrenia* and *Acremonium* were the dominant fungi genus after agarwood formation for one year, *Devriesia*, *Pseudoteratosphaeria* and *Morenoina* were the dominant fungi genus after agarwood formation for 2 years, and only *Fusarium* was the dominant fungi genus after agarwood formation for 3 years. This finding indicates that the endophytic fungi of Qi-Nan and ordinary *A. sinensis* are different. However, with the increase of induction time, the diversity and change of endophytic fungi in Qi-Nan has not been reported.

### 4.4 Antioxidant activity of Qi-Nan agarwood

Agarwood is a medicinal substance that has been used in traditional Chinese medicine. The fractions and components of agarwood from *Aquilaria* trees exhibit various pharmacological activities, including gastrointestinal regulation, neural activity, analgesic effects, cytotoxicity, and antibacterial, anti-inflammatory, antifungal, anti-diabetic, anti-asthmatic, and antioxidant activities (Wei et al., 2021). The average DPPH scavenging rate of agarwood formed by three different induction periods was 87.96%, indicating that Qi-Nan agarwood has strong antioxidant capacity. Studies have shown that chromones and sesquiterpenes are the principal components of agarwood (Chen D. et al., 2012) and have potentially pharmacological activities including antibacterial activity, AchE inhibitory, anti-inflammatory activity and antioxidant activity (Yang et al., 2014b; Li et al., 2014; Wang et al., 2016). With the increase of induction time, the content of chromones and sesquiterpenes in Qi-Nan agarwood increased, which led to the increase of active oxygen removal ability of Qi-Nan agarwood. DPPH and active oxygen scavenging trends are different, probably because DPPH is a nitrogen free radical and active oxygen is an oxygen free radical, both of which have specific reactions to different components in Qi-Nan agarwood.

## 5 Conclusion

1. In the actual production, the time of inducing the formation of Qi-Nan agarwood is not the longer the better. Although the yield of Qi-Nan agarwood increases with the induction time, the content of alcohol extract per unit weight of Qi-Nan agarwood will decrease. 2. As the same as the ordinary *A.sinensis*, xylem active cells (ray cells and interxylary phloem) are the xylem structure basis for the Qi-Nan agarwood formation. Starch is the metabolic substrate for the formation of Qi-Nan agarwood. Consume starch and soluble sugar and then Qi-Nan agarwood is formed and filled in xylem ray cells and interxylary phloem. 3. Endophytic fungal communities were different at different stages of agarwood formation in Qi-Nan agarwood and the diversity of endophytic fungal communities decreased after the formation of agarwood. Correlation analysis showed that the formation of Qi-Nan agarwood and its main components were significantly correlated with endophytic fungi. 4. Qin-Nan agarwood has strong antioxidant activity and may be further developed in the pharmaceutical industry.

## Data availability statement

The datasets presented in this study can be found in online repositories. The names of the repository/repositories and accession number(s) can be found below: https://www.ncbi.nlm.nih.gov/, PRJNA1021371.

## Author contributions

XLi: Writing – original draft. XF: Writing – review & editing, Investigation, Data curation. ZC: Writing – review & editing, Methodology. ZH: Writing – review & editing, Software, Data curation. XLiu: Writing – review & editing, Software, Data curation. GL: Writing – review & editing, Software, Data curation. HH: Writing – review & editing, Investigation. DX: Writing – review & editing, Methodology, Funding acquisition.
