# Supplementary material for: Anatomical, chemical and endophytic fungal diversity of a Qi-Nan clone of Aquilaria sinensis (Lour.) Spreng with different induction times
Source: Front Plant Sci. 2024 Mar 25;15:1320226. doi: 10.3389/fpls.2024.1320226 (PMC10999641; doi:10.3389/fpls.2024.1320226)
Supplement: Supplementary file 1 [file DataSheet_1.zip › Supplementary Figures and Tables.DOCX]

Supplementary Material

# Supplementary Figures and Tables

## Supplementary Figures


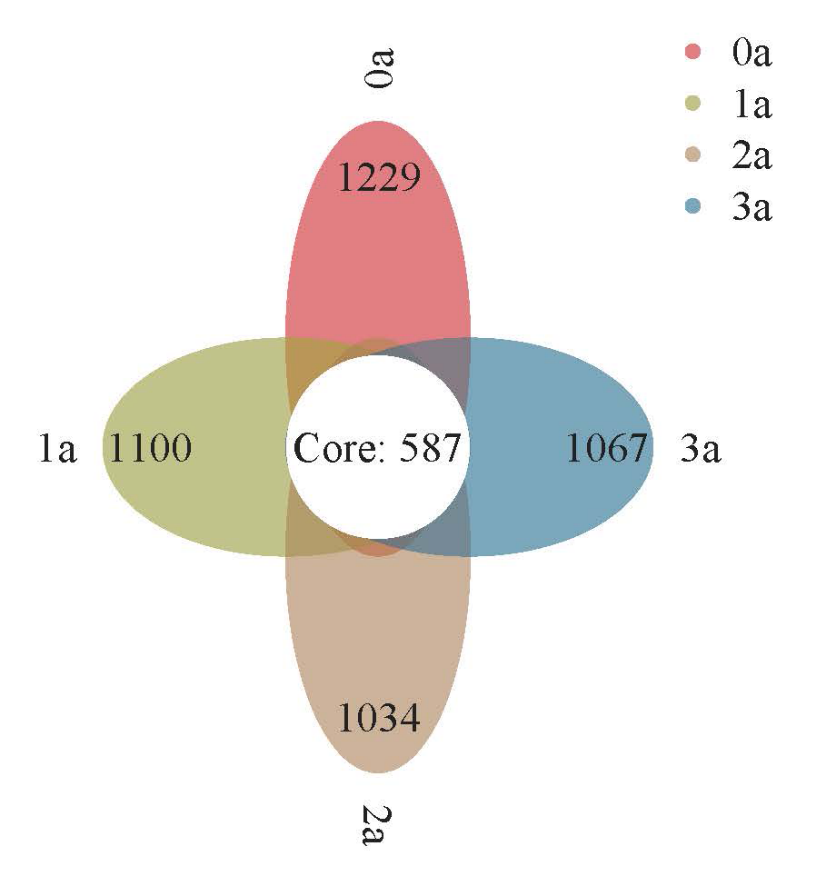


**Figure S1**. Venn diagram showing the number of specific and shared operational taxonomic units (OTUs) of endophytic fungi. 0a, Before drilling; 1a, one year after drilling; 2a, two years after drilling; 3a, three years after drilling.

## Supplementary Tables

**Table S1.** Chemical Constituents of Qi-Nan Agarwood Formed by Induction for 1, 2, 3 Years

| **No** | **Retention Time** | **Formula** | **Compounds** | **0a area**  **(%)** | **1a area**  **(%)** | **2a area**  **(%)** | **3a area**  **(%)** |
| --- | --- | --- | --- | --- | --- | --- | --- |
| 1 | 19.033 | C_15_H_24_O | Santalol, cis,.α.-** | - | 1.09 | 0.49 | 1.01 |
| 2 | 19.883 | C_5_H_10_O | Oxetane, 2,4-dimethyl-, trans-+++ | 34.02 | - | - | - |
| 3 | 21.224 | C_15_H_24_O | Aromadendrene oxide-(2)** | - | 0.07 | - | - |
| 4 | 22.161 | C_15_H_26_O | cis-Eudesm-6-en-11-ol** | - | 0.08 | 0.06 | 0.19 |
| 5 | 22.379 | C_15_H_26_O | Agarospirol** | - | 0.42 | 0.28 | 0.55 |
| 6 | 23.013 | C_15_H_24_O | Alloaromadendrene oxide-(1)** | - | 0.18 | 0.18 | 0.13 |
| 7 | 23.027 | C_15_H_22_ | 3,5,11-Eudesmatriene** | - | - | - | 0.18 |
| 8 | 23.423 | C_15_H_24_O | Spathuleno** | - | 0.05 | - | 0.1 |
| 9 | 23.873 | C_15_H_24_O | Alloaromadendrene oxide-(2)** | - | 0.12 | 0.11 | - |
| 10 | 24.384 | C_14_H_22_O | 2,4-Di-tert-butylphenol++ | 1.79 | - | - | - |
| 11 | 24.424 | C_15_H_24_ | Guaia-4,11-diene** | - | 0.06 | - | - |
| 12 | 24.718 | C_15_H_24_ | Humulene** | - | - | - | 0.11 |
| 13 | 25.843 | C_15_H_24_O | 2-(4a,8-Dimethyl-1,2,3,4,4a,5,6,7-octahydro-naphthalen-2-yl)-prop-2-en-1-ol** | - | 0.12 | 0.16 | 0.2 |
| 14 | 26.544 | C_10_H_16_O_2_ | 1-Cyclohexen-1-ol,2,6-dimethyl-, acetate*** | - | - | 0.37 | - |
| 15 | 26.773 | C_15_H_22_O | Ylangenal** | - | 0.07 | 0.34 | 0.11 |
| 16 | 26.903 | C_10_H_14_O | Thymol*** | - | 0.2 | 0.31 | 0.33 |
| 17 | 27.188 | C_12_H_14_O_4_ | 2,6-Dimethoxy-4-vinylphenyl acetate++ | 0.58 |  |  |  |
| 18 | 27.205 | C_15_H_26_O | 6-epi-shyobunol** | - | 0.03 | 0.09 | 0.1 |
| 19 | 27.642 | C_15_H_24_O | Lanceol, cis** | - | - | 0.07 | - |
| 20 | 27.765 | C_15_H_24_O_3_ | 3.α.,4.α.,9.β.,11-Diepoxymuurolan-10-ol** | - | - | 0.14 | - |
| 21 | 28.076 | C_15_H_24_O | Isoaromadendrene epoxide** | - | 0.19 | 0.11 | 0.49 |
| 22 | 28.576 | C_4_H_6_O | Cyclobutanone | 0.70 | - | - | - |
| 23 | 28.596 | C_15_H_24_O | cis-Z-.α.-Bisabolene epoxide** | - | 0.06 | - | - |
| 24 | 28.612 | C_4_H_6_O_2_ | 2-Oxetanone, 4-methyl- | 1.24 | - | - | - |
| 25 | 28.679 | C_6_H_10_O_3_ | Propyl pyruvate++ | 2.60 | - | - | - |
| 26 | 28.745 | C_8_H_14_O_3_ | Butanoic acid, anhydride++ | 8.44 | - | - | - |
| 27 | 28.821 | C_3_H_6_O_2_ | 1,3-Dioxolane++ | 4.51 | - | - | - |
| 28 | 29.592 | C_15_H_24_ | Elemene isomer** | - | - | 0.45 | - |
| 29 | 29.654 | C_9_H_16_O_5_ | Pentanedioic acid, 2-hydroxy, 1,5- diethyl ester++ | 0.25 | - | - | - |
| 30 | 29.785 | C_9_H_12_O_4_ | Phenol, 3,4,5-trimethoxy-++ | 1.92 | - | - | - |
| 31 | 30.346 | C_15_H_26_O_2_ | Aromadendrane-4,10-diol** | - | 0.03 | 0.23 | 0.13 |
| 32 | 30.715 | C_15_H_26_O | Kessane** | - | 0.22 | 0.1 | 0.55 |
| 33 | 30.888 | C_15_H_22_O | Longiverbenone** | - | 0.39 | 0.41 | 1.15 |
| 34 | 31.147 | C_15_H_24_O | Ylangenol** | - | - | 0.39 | - |
| 35 | 31.515 | C_15_H_24_O | Cedran-9-one** | - | - | - | 0.21 |
| 36 | 31.846 | C_15_H_24_O | Caryophyllene oxide** | - | 0.08 | 0.1 | - |
| 37 | 32.174 | C_9_H_10_O_4_ | Benzaldehyde, 4-hydroxy-3,5-dimethoxy-++ | 0.48 | - | - | - |
| 38 | 32.322 | C_15_H_24_ | .α.-Guaiene** | - | - | - | 0.09 |
| 39 | 32.355 | C_13_H_18_O_2_ | 4,4,5,8-Tetramethylchroman-2-ol++ | 0.57 | - | - | - |
| 40 | 32.724 | C_10_H_12_O_3_ | 2,5-Dihydroxy-4-isopropyl-2,4,6- cycloheptatrien-1-one++++ | 0.20 | - | - | - |
| 41 | 32.977 | C_15_H_24_O | α.-Santalol** | - | 0.4 | - | 0.6 |
| 42 | 33.514 | C_15_H_24_O | Isolongifolene-5-ol** | - | - | - | 0.23 |
| 43 | 33.582 | C_15_H_24_O | Longifolenaldehyde** | - | 0.1 | 0.45 | - |
| 44 | 33.775 | C_15_H_26_O | trans-Guai-11-en-10-ol** | - | 0.05 | 0.12 | 0.54 |
| 45 | 34.727 | C_15_H_22_O | 2-((2R,4aR,8aR)-4a,8-Dimethyl-1,2,3,4,4a,5,6,8a-octahydronaphthalen-2-yl)acrylaldehyde** | - | - | 0.37 | - |
| 46 | 34.815 | C_15_H_20_O_4_ | 6-Methoxyeugenyl isobutyrate++ | 0.38 | - | - | - |
| 47 | 35.907 | C_15_H_22_O_4_ | Verrucarol** | - | 0.1 | - | 0.17 |
| 48 | 36.708 | C_10_H_10_O_3_ | 4-Hydroxy-2-methoxycinnamaldehyde++ | 0.33 | - | - | - |
| 49 | 36.995 | C_10_H_12_O_4_ | 4,5-Dimethoxy-2-hydroxyacetophenone++ | 0.24 | - | - | - |
| 50 | 37.06 | C_10_H_12_O_3_ | 4-(1-Hydroxyallyl)-2-methoxyphenol++ | 7.25 | - | - | - |
| 51 | 37.073 | C_15_H_22_O | 2,3,3-Trimethyl-2-(3-methylbuta-1,3-dienyl)-6-methylenecyclohexanone** | - | 0.13 | - | 0.26 |
| 52 | 37.083 | C_15_H_24_O_2_ | (4aS,7R)-7-(2-Hydroxypropan-2-yl)-1,4a-dimethyl-4,4a,5,6,7,8-hexahydronaphthalen-2(3H)-one** | - | - | 0.08 | - |
| 53 | 37.651 | C_20_H_30_O_5_ | Andrographolide**** | - | 0.96 | 1.02 | 1.26 |
| 54 | 39.243 | C_15_H_20_O_2_ | Velleral** | - | 1.12 | 0.63 | 1.27 |
| 55 | 39.383 | C_15_H_24_O | .β.-Santalol** | - | 0.11 | 0.42 | 0.24 |
| 56 | 39.791 | C_15_H_24_O | Valerenol** | - | 0.11 | - | 0.28 |
| 57 | 45.143 | C_15_H_24_O | 4a,5-Dimethyl-3-(prop-1-en-2-yl)-1,2,3,4,4a,5,6,7-octahydronaphthalen-1-ol** | - | 0.06 | 0.07 | - |
| 58 | 49.9 | C_18_H_34_O_2_ | cis-Vaccenic acid+ | - | 0.1 | - | 0.11 |
| 59 | 50.618 | C_10_H_12_O | Benzenepropanal,.β.-methyl-++ | - | - | - | 0.41 |
| 60 | 51.678 | C_11_H_12_O_4_ | 3,5-Dimethoxy-4-hydroxycinnamaldehyde++ | 0.49 | - | - | - |
| 61 | 52.221 | C_11_H_14_O_4_ | trans-Sinapyl alcohol++ | 14.20 | - | - | - |
| 62 | 57.095 | C_17_H_14_O_2_ | 2-(2-Phenethyl)chromone* | - | 20.47 | 20.62 | 33.83 |
| 63 | 68.115 | C_18_H_16_O_3_ | 2-[2-(4-Methoxyphenyl)ethyl]chromone* | 1.75 | 38.43 | 36.88 | 37.3 |
| 64 | 68.413 | C_17_H_14_O_3_ | 2-[2-(4-Hydroxyphenyl)ethyl] chromone* | - | 8.17 | - | 0.49 |
| 65 | 71.282 | C_17_H_14_O_3_ | 2-[2-(3-Hydroxyphenyl)ethyl] chromone* | - | 0.54 | - | 0.75 |
| 66 | 72.138 | C_17_H_14_O_3_ | 6-Hydroxy-2-(2-phenylethyl)chromone* | - | 0.24 | - | 0.34 |
| 67 | 73.087 | C_18_H_16_O_4_ | 2-[2-(3-Methoxy-4-hydroxyphenyl)ethyl] chromone* | - | 20.81 | 26.43 | 13.79 |
| 68 | 73.94 | C_19_H_18_O_4_ | 2-[2-(3,4-Dimethoxyphenyl)ethyl] chromone* | - | 2.33 | 3.83 | 1.11 |
| 69 | 80.96 | C_18_H_16_O_4_ | 6-Hydroxy-2-[2-(4-methoxyphenyl)ethyl] chromone* | - | 1.28 | 2.03 | 0.83 |
| 70 | 86.709 | C_30_H_50_ | Supraene***** | 0.33 | - | - | - |
| 71 | 87.239 | C_18_H_16_O_5_ | 6-Hydroxy-2-[2-(3-methoxy-4-hydroxyphenyl)ethyl] chromone* | - | 0.84 | 1.94 | 0.24 |
| 72 | 89.459 | C_17_H_14_O_5_ | 4H-1-Benzopyran-4-one, 5-hydroxy-7- methoxy-2-(4-methoxyphenyl)-++++ | 17.71 | - | - | - |

“**” sesquiterpenes; “*” chromones; “***” monoterpenes; “****” diterpenes; “*****” Triterpenes；“+” fatty acid; “++” Aromatic compound；“+++” alkanes， “++++” Flavonoids，“-” Indicates that no modified component was detected. 0a, Before drilling; 1a, one year after drilling; 2a, two years after drilling; 3a, three years after drilling.

**Table S2** Sequencing characteristics of Qi-Nan samples

| **Sample** | **Sequences** | **Bases(bp)** | **Average Length(bp)** | **coverage** |
| --- | --- | --- | --- | --- |
| 0a-1 | 47040 | 11174067 | 237.54 | 0.996045272 |
| 0a-2 | 30443 | 7160789 | 235.22 | 0.992690603 |
| 0a-3 | 43971 | 10554826 | 240.04 | 0.99560281 |
| 1a-1 | 29546 | 7098808 | 240.26 | 0.998052813 |
| 1a-2 | 57810 | 13491152 | 233.37 | 0.997393236 |
| 1a-3 | 51674 | 12182134 | 235.75 | 0.997193547 |
| 2a-1 | 52244 | 12648705 | 242.11 | 0.996639759 |
| 2a-2 | 56756 | 13337970 | 235.01 | 0.997073258 |
| 2a-3 | 48612 | 11415235 | 234.82 | 0.995820511 |
| 3a-1 | 42664 | 9953221 | 233.29 | 0.99604187 |
| 3a-2 | 56277 | 13580165 | 241.31 | 0.996891361 |
| 3a-3 | 57600 | 13871602 | 240.83 | 0.997741748 |

Note: 0a, Before drilling; 1a, one year after drilling; 2a, two years after drilling; 3a, three years after drilling.

**Table S3** Alpha Diversity of Qi-Nan endophytic fungi communities with different induction time.

| **Alpha**  **Diversity** | **Reads** | **Richness** | **Shannon** | **Simpson** | **Pielou** |
| --- | --- | --- | --- | --- | --- |
| 0a | 39433.667±4087.672a | 887.667±10.385a | 6.863±0.100a | 0.021±0.002a | 0.701±0.011a |
| 1a | 46071.33±6974.016a | 748.667± 5.193a | 5.400±0.502a | 0.090±0.035a | 0.566±0.053a |
| 2a | 53999.000±1266.465a | 768.333±21.387a | 5.842±0.090a | 0.047±0.005a | 0.609±0.007a |
| 3a | 48904.000±3490.500a | 759.667±48.800a | 5.940±0.471a | 0.069±0.030a | 0.620±0.045a |

Note: The same lowercase letters as the column indicate that there are no significant differences between treatment. 0a, Before drilling; 1a, one year after drilling; 2a, two years after drilling; 3a, three years after drilling.

**Table S4.** Dominant order and genera (relative abundance exceeded 10 %) of endophytic fungi in Qi-Nan agarwood.

| Endophytic fungi | | 0a | 1a | 2a | 3a |
| --- | --- | --- | --- | --- | --- |
| Order | *Capnodiales* | 50.47 ± 12.77 | 29.84 ± 17.81 | 45.59 ± 15.10 | 26.29 ± 4.71 |
|  | *Pleosporales* | 12.49 ± 3.97 | 32.13 ± 2.08 | 10.67 ± 3.76 | 14.70 ± 4.15 |
|  | *Hypocreales* | 1.28 ± 0.66 | 2.29 ± 1.09 | 6.34 ± 2.29 | 22.43 ± 8.74 |
|  | *Dothideomycetes* | 1.27 ± 0.48 | 4.84 ± 1.72 | 11.33 ± 3.77 | 3.67 ± 3.52 |
|  | *Leotiomycetes* | 0.39 ± 0.31 | 12.50 ± 6.83 | 0.19 ± 0.05 | 0.22 ± 0.05 |
| Genera | *Devriesia* | 31.75 ± 5.24 | 14.48 ± 7.12 | 21.78 ± 6.71 | 8.26 ± 3.64 |
|  | *Arthopyrenia* | 5.48 ± 2.76 | 14.02 ± 2.88 | 2.82 ± 2.16 | 5.96 ± 1.63 |
|  | *Pseudoteratosphaeria* | 4.66 ± 2.81 | 3.89 ± 2.02 | 18.11 ± 3.22 | 5.41 ± 2.18 |
|  | *Acremonium* | 0.36 ± 0.24 | 12.86 ± 6.77 | 1.16 ± 0.35 | 2.52 ± 1.73 |
|  | *Morenoina* | 0.66 ± 0.17 | 4.47 ± 1.78 | 11.08 ± 3.88 | 0.56 ± 0.32 |
|  | *Fusarium* | 0.53 ± 0.36 | 1.00 ± 0.58 | 3.38 ± 1.05 | 18.71 ± 1.19 |

Note: 0a, Before drilling;1a, one year after drilling; 2a, two years after drilling; 3a, three years after drilling. Only the dominant order and genus of endophytic fungi in Qi-Nan are shown in the table.
